# Enemies with benefits: parasitic endoliths protect mussels against heat stress

**DOI:** 10.1038/srep31413

**Published:** 2016-08-10

**Authors:** G. I. Zardi, K. R. Nicastro, C. D. McQuaid, T. P. T. Ng, J. Lathlean, L. Seuront

**Affiliations:** 1 Department of Zoology and Entomology, Rhodes University, Grahamstown, 6140, South Africa; 2 CCMAR–Centro de Ciencias do Mar, CIMAR Laboratório Associado, Universidade do Algarve, Campus de Gambelas, Faro, 8005-139, Portugal; 3 The Swire Institute of Marine Science and School of Biological Sciences, The University of Hong Kong, Pokfulam Road, Hong Kong SAR, China; 4 CNRS, UMR LOG 8187, Laboratoire d’Océanologie et de Géosciences, Station Marine, BP 80, Wimereux, 62930, France

## Abstract

Positive and negative aspects of species interactions can be context dependant and strongly affected by environmental conditions. We tested the hypothesis that, during periods of intense heat stress, parasitic phototrophic endoliths that fatally degrade mollusc shells can benefit their mussel hosts. Endolithic infestation significantly reduced body temperatures of sun-exposed mussels and, during unusually extreme heat stress, parasitised individuals suffered lower mortality rates than non-parasitised hosts. This beneficial effect was related to the white discolouration caused by the excavation activity of endoliths. Under climate warming, species relationships may be drastically realigned and conditional benefits of phototrophic endolithic parasites may become more important than the costs of infestation.

Experimental and observational studies have consistently shown that parasites reduce host survival and fecundity causing a decrease in host population growth and density that can even drive the population to extinction. However, the drawbacks of being parasitized may be context-dependent. Parasites that are otherwise detrimental may become beneficial, improving host’s performance under specific environmental conditions while maintaining an overall negative effect under others (‘conditionally helpful parasites’ as defined in Fellous and Salvaudon^1^).

Significant effects of raising temperatures on populations, communities and ecosystems have been observed across many regions and in a wide variety of marine and terrestrial taxa^2^,^3^. Population extinctions and mass mortalities have been reported as a result of continuous, gradual temperature increase over relatively long temporal scales or extreme events^4^,^5^,^6^. Here we address the neglected question of whether endolithic infestation can provide a previously unrecognised protective role for mussels and increase their survival during periods of intense heat stress. Phototrophic shell-degrading endoliths (mainly cyanobacteria) bore into mussel shells, weakening shell strength; the demand for shell repair depletes energy resources at the expenses of reproduction, byssal attachment strength, general condition and growth^7^,^8^. In extreme cases, endoliths cause the shell to collapse and can be responsible for 50% of total mortality in highly infested mussel populations^7^,^8^,^9^.

Endoliths are metabolically dependent to their host; excavation into the host shell is through chemical dissolution, with the carbonate ions released being converted from calcite into CO_2_ that is then used for photosynthesis^10^. Because of their boring activity, these parasites cause a distinctive discoloration of the outer layer of the mussel shell^11^. In this study, we consider a situation in which shell-degrading endoliths could benefit their hosts. Specifically, we hypothesise that parasites lower host body temperatures during low tide aerial exposure by either enhancing solar reflectivity as a result of the whitening of the shell or by dissipating heat through photosynthetic activity. If so, we predicted that, during periods of intense heat stress, non-infested mussels would suffer higher mortality rates than those that are infested.

## Results

### Testing the effect of parasitism on mussel body temperature

**Figure 1:**
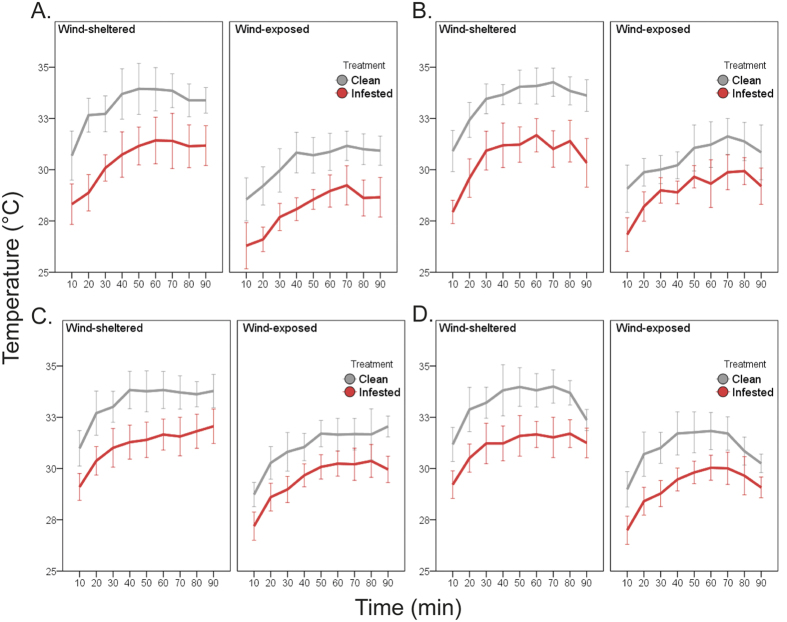
Effect of parasitism on mussel body temperatures. Results separated by trial and species: (**A**,**B**) for *Perna perna* and (**C**,**D**) for *Mytilus galloprovincialis*. Each trial was conducted separately, in different days: on the 22/01/2015 and 23/01/2015 for (**A**,**B**) and on the 29/01/2015 and 18/02/2015 for (**C**,**D**).

A general trend was observed among all trials. Regardless of the mussel bed section (i.e. wind-exposed upwind or wind-sheltered downwind) and of the time interval (i.e. 10, 20, 30, 40, 50, 60, 70, 80 or 90 minutes) considered, infested mussels always had lower body temperatures (1.9–4.5 °C) than non-infested mussels (hereafter referred to as clean mussels), even when a significant effect of the nested factor bed was detected (Fig. 1; p < 0.001, see also Supplementary Table S1; for each trial, SNK significant for a few time intervals).

In one trial of each species, the effect of treatment (clean vs. infested) was greater in downwind than upwind sections (Section × Treatment; p < 0.0001 Fig. 1b for *Perna perna* and p < 0.005 and Fig. 1c for *Mytilus galloprovincialis*).

Temperatures increased with time but mostly only for the first 30 minutes. In one *P. perna* trial, mussel body temperatures increased for the first 30 minutes in the wind-sheltered downwind section and until 20 minutes at the upwind side of the bed (Section × Time; p < 0.05 Fig. 1b). Finally, in one replicate of *M. galloprovincialis*, clean mussels showed an increase in body temperature only for 20 minutes while, in infested mussels, body temperature stabilised after 30 minutes (Time × Treatment; p < 0.05 Fig. 1d).

### Testing the effect of shell discolouration and endolithic photosynthesis on mussel body temperature

**Figure 2:**
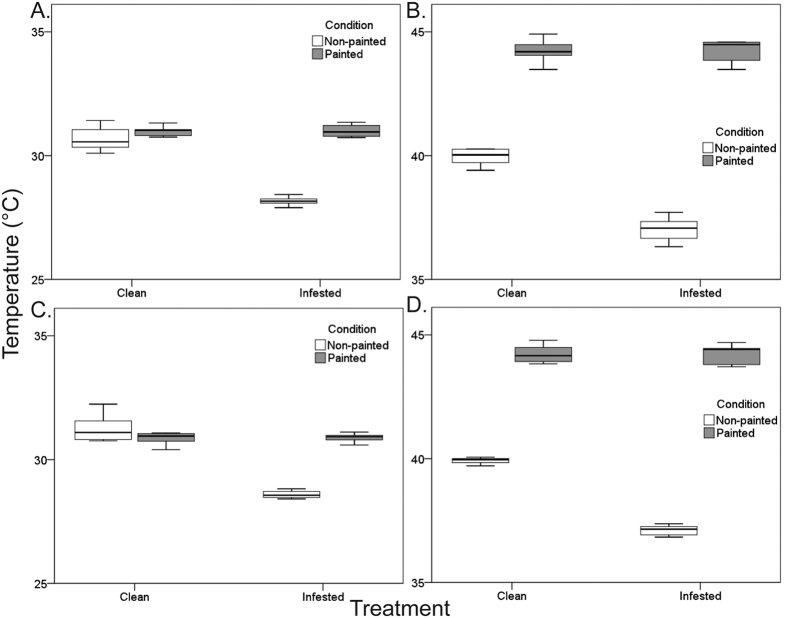
Effect of shell discolouration on mussel body temperatures. Results separated by trial and species: (**A**,**B**) for *Perna perna* and (**C**,**D**) for *Mytilus galloprovincialis*. For each species, trials were conducted separately, in different days: on the 24/01/2015 for (**A**,**C**) and 03/01/2015 for (**B**,**D**).

In both trials and for both species, body temperatures of infested mussels were significantly lower than for clean individuals, but not for the painted condition (Treatment × Condition: Pperm < 0.001 for trial 1 in *P. perna*, and p < 0.001 for all other cases, see also Supplementary Table S2; Fig. 2a,b for *P. perna*, Fig. 2c,d for *M. galloprovincialis*).

**Figure 3:**
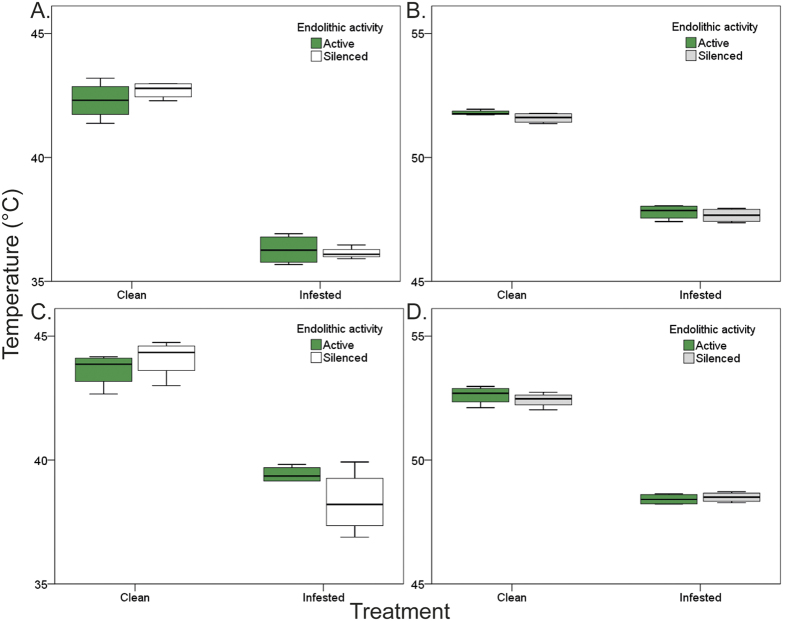
Effect of endolithic photosynthetic activity on mussel body temperatures. Results separated by trial and species: (**A**,**B**) for *Perna perna* and (**C**,**D**) for *Mytilus galloprovincialis*. Photosynthetic activity was silenced by killing microbial endoliths in boiling water. For each species, trials were conducted separately, in different days: on the 03/03/2015 for (**A**,**C**) and 04/01/2015 for (**B**,**D**).

Moreover, for biomimetic mussels, body temperatures of infested individuals were significantly lower than those of clean individuals. This was also the case for biomimetic mussels in which endoliths had been killed (silenced treatment), indicating that the treatment effect was not a by-product of endolithic photosynthetic activity (Treatment p < 0.001, see also Supplementary Table S3; Fig. 3a,b for *P. perna* and c, d for *M. galloprovincialis*).

### Testing the effect of parasitism on mussel survival during a period of extreme heat

**Figure 4:**
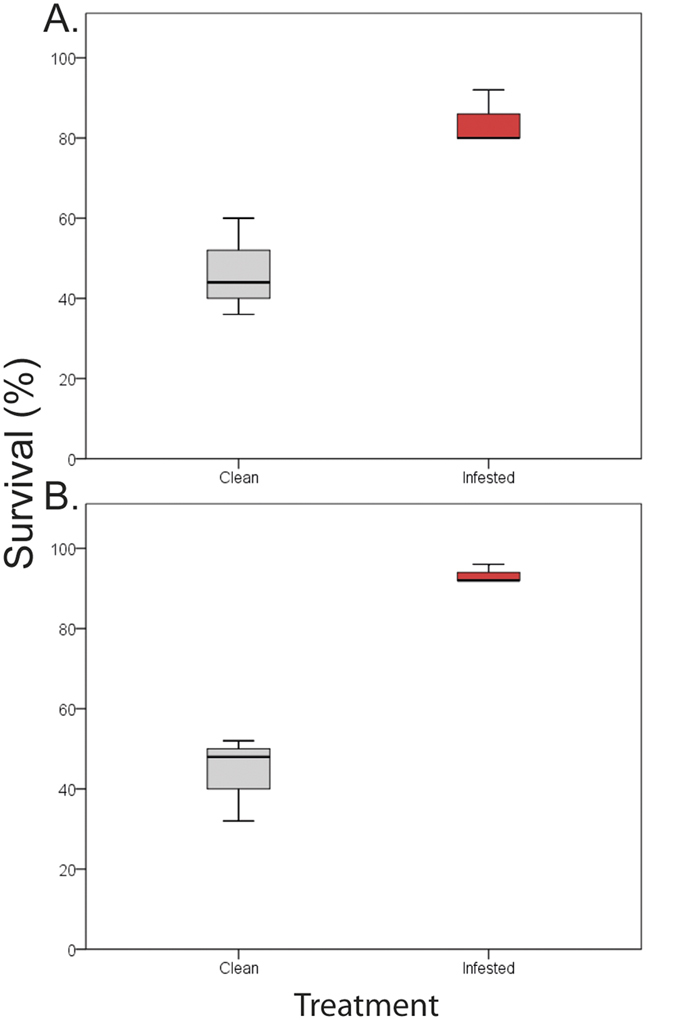
Effect of parasitism on mussel’ survival during severe heat stress. Results for (**A**) *Perna perna* and (**B**) *Mytilus galloprovincialis*.

After extreme heat, infested mussels of both species had significantly, and dramatically higher survival rates than clean mussels (Treatment p < 0.01, see also Supplementary Table S4; Fig. 4a,b for *P. perna* and *M. galloprovincialis* respectively). On average, clean mussels exhibited mortality rates 49% greater than those of infested individuals.

## Discussion

Our results unequivocally demonstrate a moderating role of phototrophic shell-degrading endoliths on the effects that extreme heat has on their hosts; endoliths substantially decreased mussel body temperatures in both wind-exposed and sheltered patches. The results also rejected the hypothesis of an active, cooling role of endolithic photosynthesis. Oxygenic photosynthetic organisms, such as vascular plants and algae, have evolved ways to cope with high light energy and reduce photo-damage reviewed in^12^. These include processes that quenche chlorophyll fluorescence in a nonphotochemical way (non-photochemical quenching, NPQ) dissipating excitation energy as heat^13^. Although the molecular mechanism for NPQ in cyanobacteria is different from the equivalent process in vascular plants and algae, it also shares similarities in serving to actively dissipate excitation energy as heat. It remains unclear which portion of energy can be dissipated through NPQ; however, because we did not detect any difference when photosynthetic activity was silenced, it is likely that the dissipation of absorbed light energy during oxygenic photosynthesis in endoliths has negligible effects on their mussel hosts. The thermal mitigation provided by the endoliths reduces the deleterious effect that heat stress has on mussel survival rates because whitening the outer shell layer increases the reflected component of solar irradiance (albedo), reducing the amount of solar energy absorbed by the shell and, as a result, the organism’s overall body temperature. The cooling mechanism is thus not a direct easing of high temperature *per se* but rather an indirect by-product of enhanced solar reflectivity.

Climate change will not involve an increase in heating through solar radiation, but during aerial exposure mussels lose heat to the surrounding air by convection, radiation and conduction. Critically, rates of heat loss through conduction are proportional to the temperature gradient between the mussel and its surroundings, which will decrease as air temperatures rise. In this situation, the effect of minimising solar heating through shell discolouration is likely to become increasingly advantageous. Gradual warming and increased frequency and duration of extreme heat-events^14^,^15^ will challenge both clean and infested individuals. As intertidal invertebrates are essentially marine species with many already living at or close to their upper thermal tolerance limits^16^, thermoregulatory discolouration caused by endolithic erosion may provide important ecological advantages. Indeed, temperature-driven mass mortalities of diverse intertidal organisms, including barnacles, limpets and mussels, have already been reported^17^,^18^.

The negative effects that shell-degrading endoliths have on their hosts in the intertidal have been assessed under a variety of environmental conditions. In particular, the emergence, persistence and strength of endolithic boring activity are largely regulated by physical stress imposed by waves or wind-borne sand particles, which enhance shell scouring^11^ and by the degree of light exposure, which favours cyanobacteria photosynthetic activity^8^.

Endolithic infestation can have lethal effects by causing shell collapse and can force critical energetic trade-offs between shell-repair and reproduction, growth and byssal attachment strength^7^,^8^. Therefore, mussels with shells weakened by endolithic erosion are more vulnerable at sites where predation is intense or during periods of strong wave action (e.g., winter storms) when shell scouring and the risk of dislodgment are both high^8^,^11^. Despite the numerous drawbacks of endolithic parasitism, our results show significant endolith-mediated heat stress protection, demonstrating that within the complex mussel-endolith relationship, both negative and positive interactions occur. Beneficial effects of endolithc algae have also been suggested for coral reefs. Coral bleaching–the loss of symbiotic dinoflagellates–is caused by climate warming and has been responsible for massive mortalities in corals. After coral bleaching, the biomass of endolithic algae rapidly increases^19^,^20^ and this may provide partial protection to the surviving symbionts from extreme radiation by decreasing the reflectivity of the skeleton^21^.

Our results, whilst showing endolith-mediated protection against thermal stress, do not measure fitness (reproductive success). Thus, the possibility that endolith positive effects might represent a transition towards mutualism remains purely speculative. It has been suggested that most mutualisms have evolved from entirely parasitic relationships and that conditionally helpful parasites represent intermediate stages in such an evolutionary transition^1^. Indeed, the nature of a symbiosis can gradually change between states depending on changes to the symbionts as well as changes in the biotic/abiotic environment in which the interaction occurs^22^,^23^.

Intertidal mussel shell colour exhibits wide plasticity, however, the distinctive shell discolouration caused by the eroding activity of endoliths goes beyond the range of such plasticity. This marked phenotypic change grants mussels higher levels of thermal resistance that are unlikely to be reached through adaptive mutations in the absence of endoliths.

The impact of environmental changes on the outcome of the mussel-endolith interaction will also depend on how temperature affects the ability of endoliths to colonize mussel shells.

Endolithic induced erosion is ubiquitous and marine calcifying organisms are globally affected by endolithic assemblages. In intertidal ecosystems, shell degrading endoliths have been reported in different bioregions, ranging from cold-temperate to sub-tropical/tropical conditions e.g. refs 9,24,25. Although microboring rates are predicted to increase as a result of future projections of ocean acidification and warming rates^26^,^27^, endolithic species or strains may individually occupy distinct ecological thermal niches e.g. ref. 28. If so, future environmental changes may alter the community structure of microborers on mussels’ shells. Indeed, such shifts in community assemblages have been reported for coralline algae and coral carbonates, experimentally exposed to increased ocean acidity and temperature regimes^26^,^29^.

Our results highlight the importance of assessing the impact of parasitic endoliths across environments and their spatial and temporal fluctuations. Further work is required to evaluate if the cooling effect of endoliths has a significant impact on mussels’ fitness. The estimation of the net result of direct and indirect endolith effects on host reproductive success is required to understand the ecological and evolutionary relevance of this interaction.

## Methods

Our study used a temperate (*Mytilus galloprovincialis*) and a subtropical (*Perna perna*) intertidal mussel species as host organisms. Phototrophic endoliths parasitizing these mussels are predominantly *Hyella* spp., *Plectonema* spp. and *Mastigocoleus testarum*^9^,^11^.

Field experiments and mussel collections were carried out on the south coast of South Africa (Jongensfontein 34°25′12.26″S, 21°21′28.27″E; Old Womans River 33°28′56.54″S, 27°09′4.84″E; Kenton-On-Sea 33°41′29.86″S, 26°40′26.00″E) where these mussel species represent dominant constituents of intertidal communities, co-occur in mixed populations and can be highly infested by endoliths reviewed in ref. 30. All replicate trials were conducted with fresh individuals of 4–5 cm (±0.5) in shell length, that were collected within 48h of experimental set up and, prior to each experiment, kept in a controlled environment chamber (submerged in seawater at 19 °C, under a 12:12 h light: dark regime).

Body temperature of mussels was investigated using infrared thermography (IRT), which has been increasingly used to assess the body temperature of intertidal organisms non-invasively reviewed in ref. 31. IRT measures of shell surface temperature of intertidal molluscs has been shown to be a reliable proxy for body temperature^32^. Strong, significant positive correlation (r^2^ = 0.96, n = 122, p < 0.001, y = 0.9722x + 1.1239^33^) between these two mussel species’ body temperature (measured with digital thermocouples) and their shell surface temperature has recently been found in the field for our intertidal study system, over a wide range of temperature values (from 22 °C to 40 °C).

Within each experiment, all infrared images were taken using either a Fluke Ti20 (Fluke Corporation, Everett, USA) or a Testo 875-1iSR (Testo AG, Germany) hand-held infrared thermometer with emissivity values set at 0.95, a thermal sensitivities of 0.09 °C (Fluke Ti20) or 0.05 °C (Testo 875-1iSR), and accuracies of ±2 °C or ±2% of reading, whichever was greatest see e.g. refes 31,34. All images were acquired at approximately 1.5 m from ground level and during air exposure, high sun elevation (i.e. between 10:00 to 14:00 local time) and sunny austral summer days (between 15 January and 15 March, 2015).

### Testing the effect of parasitism on mussel body temperature

**Figure 5:**
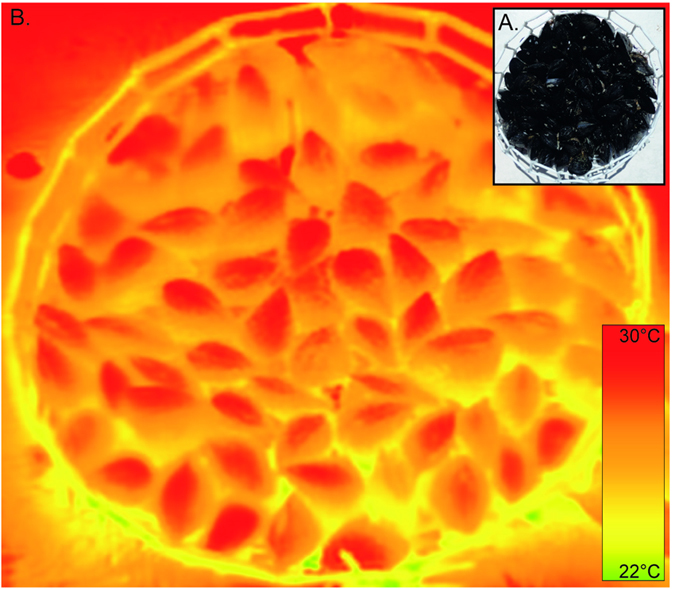
Example of an artificial mussel bed. Digital photograph of the bed (**A**) and its thermal infrared image (**B**).

To determine the effect of parasites on body temperatures of mussels in aggregation, artificial mussel beds were made using live mussels. For each species, beds with either non-infested or infested mussels were assembled (n = 3 per treatment; Fig. S2). These beds were circular (diameter of ca. 15 cm) and made up of mussels (ca. 55) arranged vertically to mimic their position when naturally aggregated. Partially rigid, white PVC net (mesh size 4 cm) was placed around and under the beds to keep the mussels in position see Fig. 5; methodology adapted from^35^. Bed treatments were positioned on white tiles and aerially exposed to ambient, natural conditions for one and a half hours during which IR images were taken every 10 minutes.

For each species, two replicate trials with new individuals were conducted on different days.

### Testing the effect of shell discolouration and endolithic photosynthesis on mussel body temperature

To determine whether the effect of parasites on body temperatures was caused by the white discolouration of the shell, mussels (n = 6 each species and treatment) were painted black with a permanent marker just before the trial. All mussels were aerially exposed and placed on white tiles in a solitary position (i.e., oriented vertically to the substratum with the ventral side facing the tiles) and equidistant from each other (approximately 10 cm). IR images were taken every 10 minutes for one and a half hours.

To test whether endolithic photosynthetic activity caused reduced mussel body temperatures by intercepting light energy, IR images of biomimetic mussels made with shell valves with live or silenced (dead) endoliths were compared. Biomimetic mussels were built to mimic the thermal characteristics of living mussels adapted from^36^. They were filled with marine silicone sealant and left to dry at room temperature for 48h prior to the experiments. Endoliths were killed (hence photosynthetic activity silenced) by keeping the mussel shells in boiling water for an hour before assembly them.

For each species, both experiments were repeated twice with new individuals and on different days.

### Testing the effect of parasitism on mussel survival during a period of extreme heat

To determine the effect of parasites on survival of aggregated mussels after periods of intense heat stress, mussels were translocated to bare patches within the mussel zone and survival rates of the different treatments (i.e., infested and non-infested) were compared at the end of a period of extreme heat. The experiment was set up within the natural intertidal mussel zone at Old Womans River on February 24^th^ 2015, seven days before predicted extremely high air temperatures for a nearby (ca. 20 km) coastal weather station [at the World Meteorological Organization (WMO) weather station 68843, Port Alfred Airport]. The experiment ran for two weeks; during this period air temperature reached maximum values for the year (September 2014-September 2015; see also Supplementary Fig. S1). High summer temperatures have often been reported as having catastrophic effects on intertidal communities, including mass mortality of many species^37^,^38^, thus providing excellent conditions to test our hypothesis.

Each translocated patch of mussels was secured to the rock by a metal, quadrat frame (15 × 15 cm) attached to the shore with screws and orientated perpendicularly to the incoming tide. For each species, treatments were haphazardly assigned to three quadrats and each replicate was made up of 25 mussels. To avoid mortality due to factors other than heat stress (e.g., wave action or predation), plots were covered tightly with soft white plastic 2 cm mesh.

The experiment was ended and final survival rates were recorded when LT_50_ was reached in at least two plots, i.e. on the 10^th^ and 15^th^ of March for *M. galloprovincialis* (temperate species) and *P. perna* (subtropical species) respectively.

### Data analyses

For each experiment, IR images were analysed either using IRSoft 3.1^39^ or SmartView 3.2.639.0^40^. As even weak air movement was found to influence shell temperatures in the IR images, each image of an artificial mussel bed was considered as two sections based on prevailing wind conditions: a wind-exposed upwind and a wind-sheltered downwind section. An external circular buffer of 1 cm radius and an internal corridor of 1 cm was manually drawn in each image to exclude edge effects of both the bed and between sections^35^. Within each zone of interest, seven mussels were haphazardly selected at each time interval and their temperatures recorded using one point-measure in the middle of each mussel.

Separate analyses were done for each trial and species. The effect of parasites on mussel body temperature was tested using a four-way ANOVA with section (wind-exposed upwind or wind-sheltered downwind), time (10, 20, 30, 40, 50, 60, 70, 80 or 90 minutes), treatment (non-infested or infested) as orthogonal factors and bed (1, 2 or 3) as the nested random factor.

The effect of white discoloration or of endolithic activity on mussel body temperature was tested with a two-way ANOVA with treatment (non-infested or infested) and condition (painted or non-painted; active or silenced) as orthogonal fixed factors and the average of all measures taken for each mussel as the dependent variable in each analysis. Finally, the effect of parasites on mussels’ survival rates was tested using a one-way ANOVA with treatment (non-infested or infested) as orthogonal factor.

Prior to analyses, data were tested for homogeneity of variances with Cochran’s test. All ANOVA analyses were done using GMAV^41^. When parametric assumptions were not met even after standard transformations, PERMANOVA standalone software^42^ was used instead on untransformed data. Student-Newman-Keuls (SNK; for ANOVA) test or pairwise tests (for PERMANOVA) were run for significant sources of variation. The α-values in the pairwise tests were adjusted using Bonferroni correction to reduce type I errors in multiple comparisons.

## Additional Information

**How to cite this article**: Zardi, G. I. *et al*. Enemies with benefits: parasitic endoliths protect mussels against heat stress. *Sci. Rep.* **6**, 31413; doi: 10.1038/srep31413 (2016).
